# A long non-coding RNA *lncRNA18313* regulates resistance against cadmium stress in wheat

**DOI:** 10.3389/fpls.2025.1583758

**Published:** 2025-06-02

**Authors:** Sujing Zhao, Hongxia Bai, Ziyi Fan, Mo Zhu, Zongbo Qiu

**Affiliations:** ^1^ College of Life Science, Henan Normal University, Xinxiang, China; ^2^ The Observation and Research Field Station of Taihang Mountain Forest Ecosystems of Henan Province, Henan Normal University, Xinxiang, China

**Keywords:** long non-coding RNA, wheat, Cd stress, RNA-Seq, heavy metal

## Abstract

Long non-coding RNAs (lncRNAs) have been demonstrated to play key roles in plant response and adaptation to heavy metal stresses. However, the exact biological functions and potential regulatory mechanism, especially in wheat’s response to cadmium (Cd) stress, are still poorly understood. We have previously discovered a Cd stress-related lncRNA in wheat, namely *TalncRNA18313*. In this study, qRT-PCR analysis revealed that *TalncRNA18313* was expressed extensively in wheat leaves, and its accumulation was highly induced by Cd stress. To further fully explore the function of *lncRNA18313* in response to Cd stress, *lncRNA18313* was cloned from wheat (*Triticum aestivum* L.), and was transformed into *Arabidopsis*. When *TalncRNA18313* was heterologous expressed in *Arabidopsis*, the transgenic plants exhibited enhanced Cd tolerance characterized by lower malondialdehyde (*MDA*) levels and higher activities of key antioxidant enzymes, such as catalase (*CAT*), superoxide dismutase (*SOD*) and peroxidase (*POD*). Subsequently, RNA-sequencing (RNA-seq) analysis demonstrated that 370 genes were differentially expressed in *lncRNA18313* overexpressing transgenic lines under Cd stress comparing to wild type plants. Among the genes regulated by *lncRNA18313*, the most significantly enriched were those involved in transcriptional regulation and antioxidative defense responses. These results suggest that *TalncRNA18313* plays a crucial role in improving Cd tolerance in wheat by modulating key stress-related pathways, particularly those critical for coping with oxidative damage and regulating gene expression under Cd stress. This discovery contributes to the expanding understanding of knowledge about the involvement of lncRNAs in plant stress responses and offers promising potential for improving crop resilience to environmental stresses.

## 1 Introduction

Heavy metal pollution has emerged as one of the most severe and widespread environmental challenges in China, driven by the rapid expansion of modern industries, agriculture, and urbanization (Li et al., 2024). Among these heavy metals, cadmium (Cd) is particularly concerning due to its extremely toxic and highly mobile in natural environment. It readily accumulates in crop plants, posing a significant threat to both agricultural productivity and human health (Cao et al., 2023; Zhang et al., 2023). As a widely consumed crop globally, wheat (*Triticum aestivum* L.) has become a major and progressively growing source of Cd intake for humans, as it can enter the human body through the food chain, thereby posing considerable health risks (Zhu et al., 2023; Zhang et al., 2024). In addition to its harmful effects on human health, excessive Cd accumulation in wheat significantly disrupts plant growth, morphology, physiology, and metabolism, both directly and indirectly. This results in substantial losses in wheat yield and quality (Thind et al., 2020; Zhu et al., 2022; Rasheed et al., 2024). Therefore, it is imperative to dissect the regulatory mechanisms involved in Cd detoxification to develop effective strategies to reduce excessive Cd into wheat. Plants have evolved various defense mechanisms to detoxify Cd toxicity, including chelation, transportation and vacuolar sequestration, which help restricts Cd accumulation and minimize its harmful effects (Cao et al., 2023; Wang et al., 2025). Previous studies on stress gene regulation have primarily focused on identifying and analyzing of specific protein-encoding genes associated with Cd homeostasis and detoxification, such as key Cd absorption genes and various heavy metal transporters (Cao et al., 2023; Rasheed et al., 2024). However, the underlying molecular mechanism responsible for Cd stress in wheat is still not fully explored.

In recent years, long non-coding RNAs (lncRNAs) have become recognized as key regulators of plant growth, governing a variety of essential biological processes (Liu et al., 2012; Zhao et al., 2023). LncRNAs are transcripts longer than 200 nucleotides, typically lacking or having no capacity of protein-coding potential (Wierzbicki et al., 2021). A growing body of evidence indicates that lncRNAs play a crucial role in regulating gene expression in plants through a variety of complex mechanisms, such as interaction with hormones and transcription factors, as well as involvement in alternative splicing (Bardou et al., 2014; Liu et al., 2015).

Along with the advancement of next-generation RNA sequencing and recent improvements in bioinformatics tools, an increasing number of lncRNAs have been identified and confirmed to play a pivotal role in orchestrating plants’ responses to varying environmental stresses. These include drought stress in wheat (Li et al., 2022) and cassava (Ding et al., 2019), cold stress in wheat (Lu et al., 2020) and *Brassica napus* (Waseem et al., 2022), alkaline stress in wheat (Wei et al., 2022) and sugar beet (Zou et al., 2020), lead (Pb) stress in poplar (Chen et al., 2022), aluminum stress in *Medicago truncatula* (Gui et al., 2022), and Cd stress in rice (Chen et al., 2018) and *Brassica napus* (Feng et al., 2016). Moreover, several plant lncRNAs have been studied intensively. For instance, a salt-responsive lncRNA, *lncRNA973*, was identified in cotton, which was induced by salt stress. Transgenic *Arabidopsis* overexpressing *lncRNA973* could facilitate salt tolerance by modulating genes involved in antioxidant defense, transcription factors and photosynthesis (Zhang et al., 2019). In *Betula platyphylla*, two Cd-responsive lncRNAs, *lncRNA28068.1* and *lncRNA30505.2*, have been shown to increase Cd tolerance by modulating the expression of their target genes, heat shock protein (HSP18.1) and L-lactate dehydrogenase A (LDHA) (Wen et al., 2020). In our previous study, we identified 10,044 lncRNAs in the roots of wheat that responded to Cd stress using RNA-seq technology. Among these, 377 lncRNAs were differentially expressed in response to Cd stress. Furthermore, we found that overexpressing *lncRNA37228* could confer Cd tolerance in *Arabidopsis* (Zhu et al., 2023). Although many Cd-responsive lncRNAs had been recognized or predicted in wheat, there may be a large number of lncRNAs whose biological functions have not yet been characterized, warranting further investigation.

The lncRNA showing the highest induction in response to Cd stress, namely *lncRNA18313*, in the wheat genome was identified in our previous research (Zhu et al., 2023). However, the biological function of *lncRNA18313* in Cd stress response remained elusive. Here in this study, we cloned *lncRNA18313* and examined its expression levels in wheat with and without Cd stress exposure. Subsequently, overexpression of *lncRNA18313* in transgenic *Arabidopsis* plants demonstrated its positive role in enhancing Cd stress tolerance. To further explore the underlying mechanisms, transcriptome sequencing of the overexpressed *lncRNA18313* plants and the control plants was conducted to investigate differentially expressed genes in response to Cd stress. These findings not only provide insights into the genetic and molecular mechanisms by which *lncRNA18313* mediates Cd stress response in wheat but also lay a theoretical foundation for genetic improvement strategies aimed at enhancing Cd tolerance in plants.

## 2 Materials and methods

### 2.1 Plant materials and CdCl_2_ treatment

Wheat cultivar ‘Zhengmai 366” was kindly provided by Henan Academy of Agricultural Sciences. Seeds, surface-sterilized, were firstly germinated at 26°C for 2 days. The germinated seeds were subsequently transferred to Petri plates (16 cm in diameter) and cultivated in an illumination incubator with a day/night temperature of 25°C/16°C, 65%-75% relative humidity, and a 12-hour light/dark cycle (light intensity: 750 μmol m^-^² s^-^¹). When the seedlings were 10 days old (with two fully expanded leaves), uniform seedlings were transferred to a nutrient solution supplemented with 100 μM CdCl_2_·2.5H_2_O for 7 days. At 0, 1, 3, 5 and 7 days of CdCl_2_ stress, roots, stems and leaves were harvested separately, rapidly frozen in liquid nitrogen, and stored at -80°C.

Seeds of wild-type (WT) and transgenic plant were sown on plates containing half-strength Murashige and Skoog (1/2 MS) solid medium in the dark at 4°C for 2 days. Afterward, the plates were moved to a growth chamber with long-day conditions (16-hour photoperiod and light intensity of 120 μmol m^-2^ s^-1^) and maintained at 22°C for 8 days. Then, 8-day-old seedlings exhibiting uniform growth were transplanted into soil and placed back in a growth chamber under the same conditions. For Cd treatment, six-week-old WT and transgenic plants (with fully expanded rosette leaves) grown in soil were divided into two groups: control and Cd treatment. The plants were irrigated with 1/2 MS solution, with or without 100 μM CdCl_2_·2.5H_2_O, for 4 days. Each experimental group comprised at least three plants, and all treatments were conducted with three biological replicates. After 4 days of Cd treatment, rosette leaves were harvested, immediately frozen in liquid nitrogen, and stored at -80°C.

### 2.2 Vector construction and genetic transformation

A 414 bp full-length genomic sequence of *lncRNA18313* was amplified by PCR from genomic DNA extracted from ten-day-old wheat seedlings using the primers 5′- TGCTCTAGA TCCGCTATTCCGATG -3′ and 5′- GGGGTACC GTTGGATAGGCT -3′. Sequencing confirmed PCR fragments were successfully inserted into pCAMBIA1300 expression vector driven by the cauliflower mosaic virus (CaMV) 35S promoter, using the restriction endonuclease XbaI and KpnI. These recombinant vectors were introduced into *Agrobacterium* EHA105 strain, and subsequently used to transform *Arabidopsis* (ecotype Columbia 0) through floral dip method as previously reported (Clough and Bent, 1998). Transgenic lines expressing *lncRNA18313* were screened using 50 mg L^-1^ kanamycin and then further confirmed by PCR amplification. Subsequently, physiological and biochemical characterization, gene expression analysis, and RNA sequencing were performed on T_3_ homozygous lines unless otherwise specified.

### 2.3 RNA extraction and gene expression analysis

Total RNAs were extracted from wheat seedlings or *Arabidopsis thaliana* using Trizol reagent (Tiangen, Beijing, China) following the manufacturer’s instructions and M5 SuperFast plus qPCR RT kit (Tiangen, Beijing, China) was used for first-strand cDNA synthesis. Relative expression levels of *lncRNA18313* and candidate genes were analyzed with qRT-PCR and elongation factor (*TaEF-1α*) as the reference gene was normalized gene expression using the 2^-ΔΔCt^ method, as described by Livak and Schmittgen (2001). The primers used in the experiment are shown in **Supplementary Table S1**. Each sample was conducted with three biological replicates.

### 2.4 Transcriptome analysis

Rosette leaves from six-week-old wild-type and transgenic *Arabidopsis thaliana* (T_3_ generation) grown under Cd stress (100 μM CdCl_2_, 4 d) were collected and sent to Lianchuan Biological Technology Co. Ltd. (Hangzhou, China) for RNA sequencing (RNA-seq). Each sample included three biological replicates, with each replicate consisting of pooled leaves from six independent plants. For bioinformatics analysis, all raw reads that passed quality control in FastQC were trimmed to eliminate low quality sequences, and mapped to the *Arabidopsis* reference genome using HISAT2. Reads normalized expression level were quantified using RSEM (v1.2.8) to calculate FPKM values and differential expression analysis was evaluated using DESeq2 and edgeR package (version 1.20.0). Genes with *p* value ≤ 0.05 and | log_2_ (fold change) | ≥ 2 were defined as differentially expressed genes (DEGs). Gene ontology (GO) and Kyoto Encyclopedia of Genes and Genomes (KEGG) enrichment analysis of DEGs were conducted using GOseq R packages and KOBAS 2.0 software, respectively.

### 2.5 Determination of *MDA* content and antioxidant enzyme activity

Rosette leaves from six-week-old seedlings of WT and transgenic *Arabidopsis* grown under normal and Cd stress conditions (100 μM CdCl_2_) for 4 days were collected to quantify malondialdehyde (*MDA*) contents and antioxidant enzyme activity. *MDA* concentration was assessed using the thiobarbituric acid (TBA) method as described previously (Zhu et al., 2022). *SOD* activity was assessed by monitoring the inhibition of nitroblue tetrazolium at 560 nm, following the method described by Giannopolitis and Ries (1977). *CAT* activity was evaluated at 240 nm by observing a decrease in absorbance due to H_2_O_2_ degradation (Aebi, 1984). *POD* activity was assayed using guaiacol and hydrogen peroxide as substrates, with the reaction monitored at 470 nm (Zhang and Kirham, 1994).

### 2.6 Statistical analysis

The data was presented as mean ± SE based on three independent replications. One-way analysis of variance (ANOVA) was carried out to assess significance differences between the control and treatment groups, followed by Duncan’s multiple range test (*p* < 0.05).

## 3 Results

### 3.1 Characteristics and expression analysis of *TalncRNA18313*

**Figure 1:**
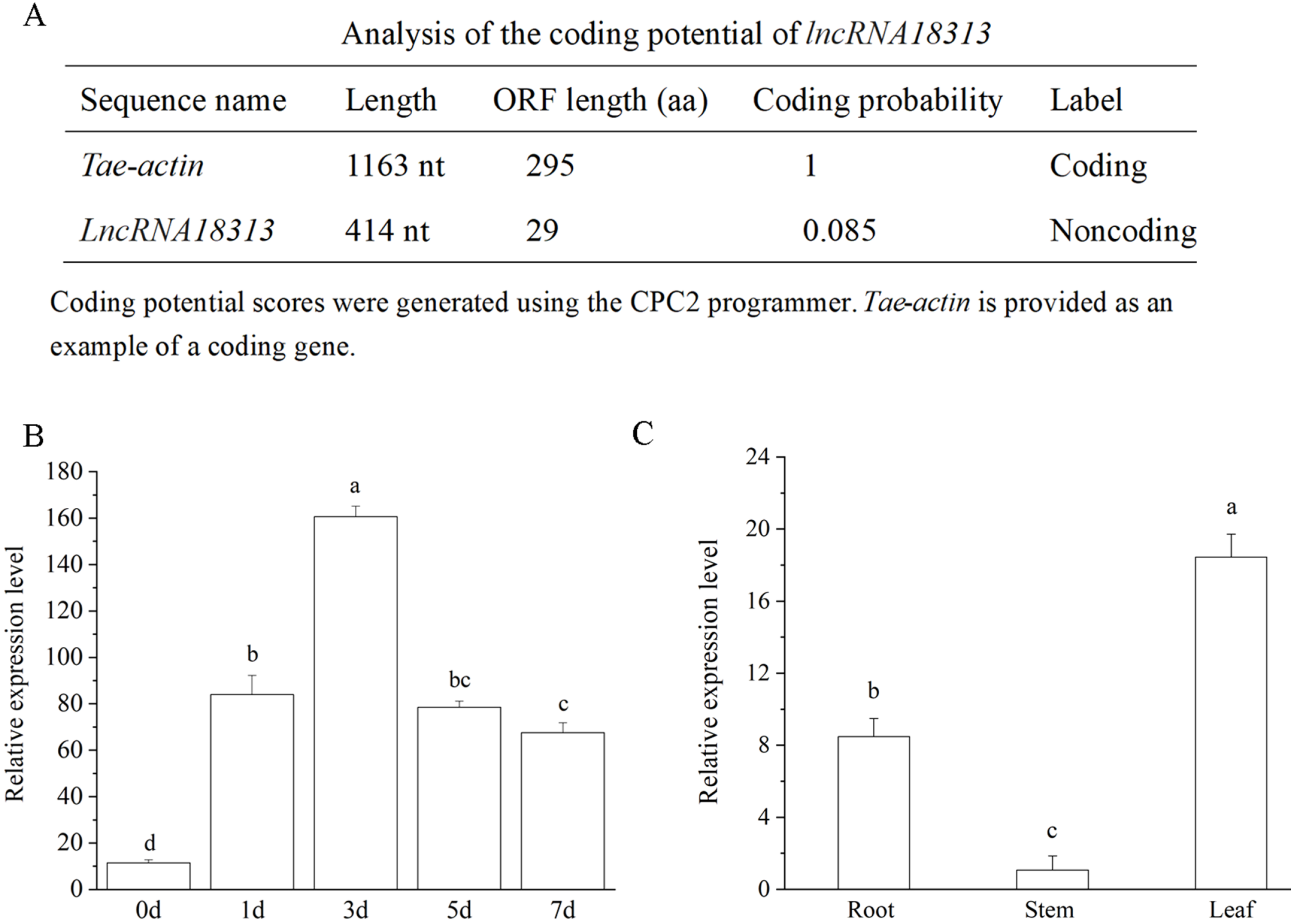
Characteristics and expression analysis of *TalncRNA18313*. **(A)** Analysis of the coding potential of *lncRNA18313*. **(B)** The expression levels of *lncRNA18313* in wheat leaves treated with 100 μM CdCl_2_ at different time points. **(C)** The expression levels of *lncRNA18313* in different tissues of wheat. Gene expression was measured by qRT-PCR, with the expression of *lncRNA18313* normalized against the *TaEF-1α* gene. Each bar represents the mean ± standard error of six replicates. Columns labeled with different letters indicate a significant difference (*p <*0.05), as determined by Duncan’s multiple range test.

In our previous study, 242 lncRNAs responsive to Cd stress were identified in wheat through RNA sequencing (RNA-Seq) (Zhu et al., 2023). Among dozens of identified Cd-responsive lncRNAs, *lncRNA18313* was strongly induced by Cd stress in wheat. Therefore, *TalncRNA18313* was chosen for further analysis. The sequence of *TalncRNA18313* was cloned from wheat variety ‘Zhengmai No. 366’, with the sequence information provided in **Supplementary Table S1**. The length of *TalncRNA18313* is 414 nucleotides, with the maximum open reading frames (ORFs) encoding 29 amino acids. However, it was predicted to lack protein-coding potential, as indicated by the Coding Potential Calculator (CPC) (**Figure 1A**). Given the low conservation of lncRNAs, the sequence of *TalncRNA18313* was subjected to NCBI BLAST, but no significant homologous sequences were identified in *Arabidopsis* or other plants species.

To elucidate the role of *lncRNA18313* in Cd stress, we firstly used qRT-PCR to explore the expression of *lncRNA18313* at different time points in wheat treated with 100 μM CdCl_2_ treatment. As shown in **Figure 1B**, the expression of *lncRNA18313* in leaves was rapidly up‐regulated, reaching its peak at the 3th day after Cd treatment, then the expression of *lncRNA18313* declined but remained at a higher expression level in contrast to that the seedlings under normal growth conditions (without Cd application). In agreement with our RNA-Seq data, the expression of *lncRNA18313* was upregulated by 16.35-fold in wheat leaves after 3 days of Cd treatment. Additionally, no significant variation in *lncRNA18313* expression was detected at different time points under normal growth conditions (**Supplementary Figure S1**). Those results suggested that *lncRNA18313* was involved in wheat responsive to Cd stress.

We also performed qRT-PCR to assess the expression levels of *lncRNA18313* in the leaves, stems and roots of wheat. Notably, a relatively high level of *lncRNA18313* expression was found in the wheat leaves (**Figure 1C**), while its transcript level in the stem was very low, suggesting that *lncRNA18313* exhibited highly tissue-specific expression patterns.

### 3.2 *TalncRNA18313* overexpression positively regulates Cd tolerance in *Arabidopsis*

**Figure 2:**
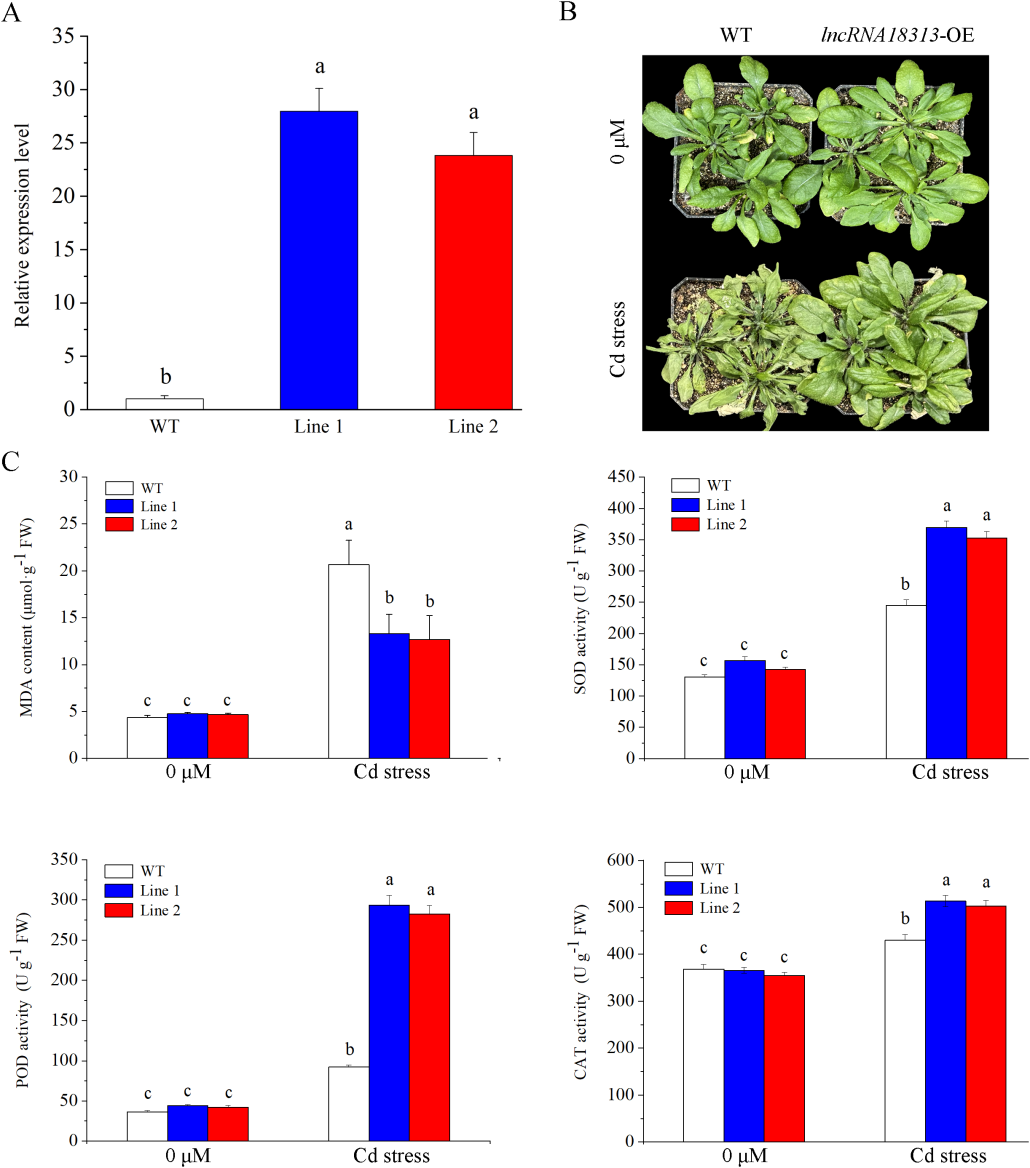
Overexpression of *TalncRNA18313* confers Cd tolerance in *Arabidopsis*. **(A)** Expression levels of *TalncRNA18313* in wild-type (WT) and *lncRNA18313*- overexpressing (OE) *Arabidopsis* lines. The expression of *TalncRNA18313* was normalized to the *AtActin* gene. **(B)** Phenotypes of rosette leaves in 6-week-old WT and *TalncRNA18313*-OE *Arabidopsis* seedlings exposed to 0 μM and 100 μM CdCl_2_ for 4 days. **(C)** Determination of malondialdehyde (*MDA*) content and the activities of superoxide dismutase (*SOD*), peroxidase (*POD*), and catalase (*CAT*) in WT and *TalncRNA18313*-OE lines under 100 μM CdCl_2_ treatment for 4 days. Each bar represents the mean ± SD for three biological replicates. Columns labeled with different letters indicate significant differences (*p* < 0.05), as determined by Duncan’s multiple range test.

To directly characterize the role of *TalncRNA18313* in plant response to Cd stress, transgenic *Arabidopsis* overexpressing *TalncRNA18313* transcript were generated and two independent lines were randomly selected for further analysis. qRT-PCR analysis showed that the expression levels of *lncRNA18313* in transgenic line 1 and 2 were approximately 20-fold higher than in the wild-type (WT) (**Figure 2A**), indicating that the transgenic lines overexpressing *TalncRNA18313* had been successfully generated and could be used for further investigation.

To decipher the physiological functions of *TalncRNA18313* in response to Cd stress, 6-week-old WT and *lncRNA18313*-OE *Arabidopsis* seedlings were treated with 100 μM CdCl_2_ in growth chambers. After 4 days of Cd treatment, WT plants exhibited severe damage, with most leaves completely withered and dry. In contrast, *lncRNA18313*-OE plants showed less damage, exhibiting fewer wilted leaves and maintaining a relatively healthy appearance compared with WT plants (**Figure 2B**). We thus quantified malondialdehyde (*MDA*) content as an indicator of membrane system damage. Consistent with the observation of leaf phenotypes, *MDA* contents in the leaves of *lncRNA18313*-OE lines were notably lower compared to those in WT plants (**Figure 2C**), suggesting that overexpression of *TalncRNA18313* resulted in less oxidative damage under 100 μM CdCl_2_ stress. As previously reported, antioxidant enzymes are crucial for scavenging reactive oxygen species (ROS) during the initial stages of Cd stress. In an agreement, Cd stress led to a significant increase in the activities of *SOD*, *CAT* and *POD* in both WT and *lncRNA18313*-OE lines. However, after 4 days of Cd stress, the activities of these enzymes were notably higher in the *lncRNA18313*-OE lines compared to WT plants (**Figure 2C**), demonstrating an enhanced capacity for ROS scavenging in the transgenic plants. These findings indicate that *TalncRNA18313* plays a vital role in mitigating Cd-induced oxidative damage, thereby improving plant tolerance to heavy metal stress.

### 3.3 Analysis of transcriptome sequencing data and identification of DEGs

**Figure 3:**
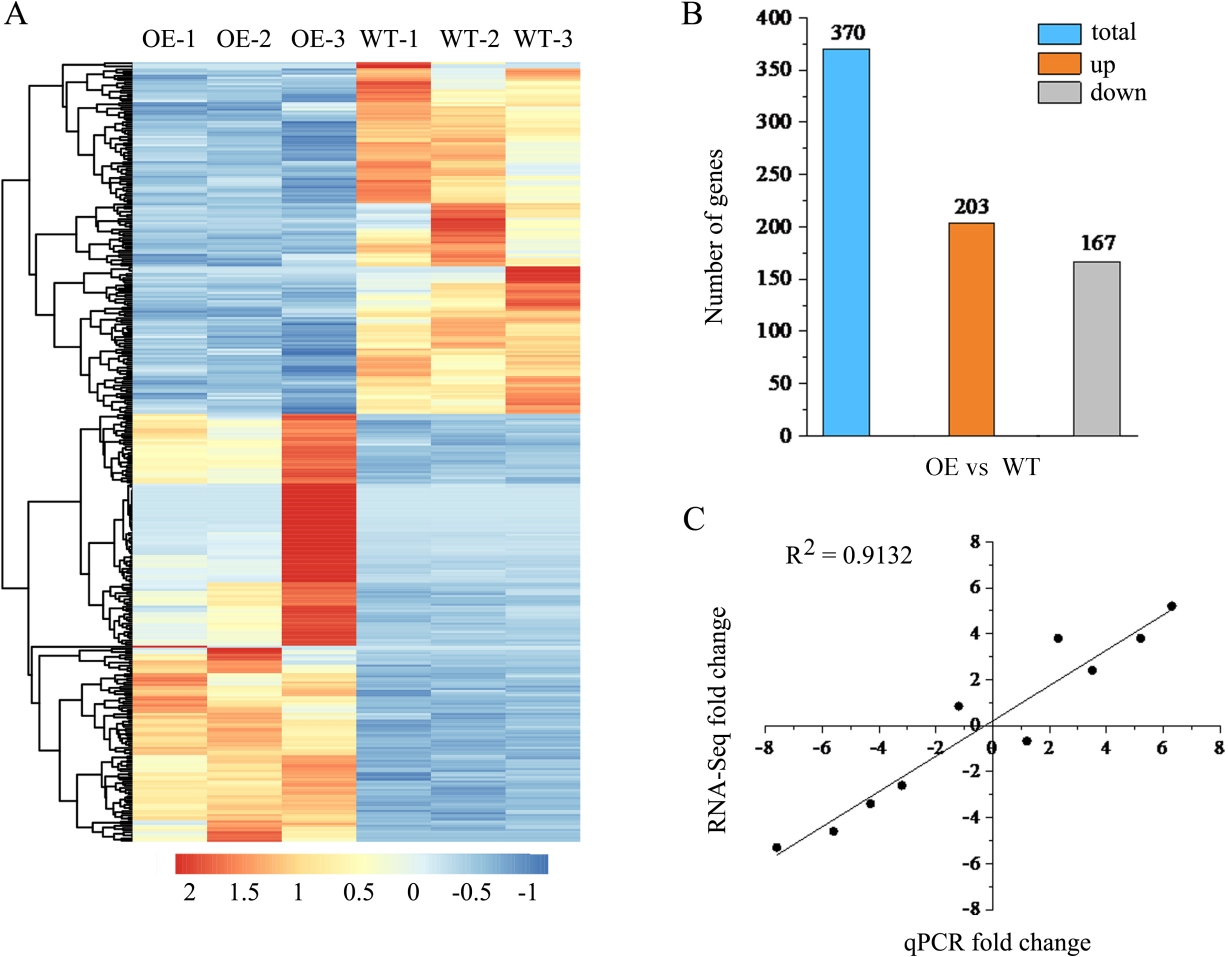
RNA-seq analysis of differentially expressed genes (DEGs) in *lncRNA18313*-OE and WT plants under 100 μM CdCl_2_ conditions for 4 days. **(A)** Hierarchical clustering of all DEGs between WT and *lncRNA18313*-OE under Cd stress, based on log_10_ RPKM (number of fragments per kilobase of transcript per million fragments mapped) values. The color scale (from blue to red) represents gene expression level from low to high. **(B)** Changes in DEGs between *lncRNA18313*-OE and WT plants under Cd stress. The number of up- and down-regulated genes between *lncRNA18313*-OE and WT is summarized. **(C)** Correlation between RNA-seq (y-axis) and qRT-PCR (x-axis) data, with the assay conducted for 10 randomly selected DEGs.

To further obtain potential genes regulated by *lncRNA18313* in Cd tolerance, RNA sequencing (RNA‐seq) was performed on 6-week-old wild-type (WT-1, WT-2 and WT-3) and *lncRNA18313*-OE lines (OE-1, OE-2 and OE-3) in order to assess transcriptional changes under Cd stress conditions. Six libraries were constructed from the rosette leaves of WT and *lncRNA18313*-OE lines, with three biological replicates for each line and 5 plants per replicate. Each library contained over 5.08 Gb of clean bases, with a Q20 percentage exceeding 99.76%, a Q30 percentage over 98.78%, and a GC content ranging from 44.5 to 45.5% (**Table 1**). The clean data were subsequently aligned to the *Arabidopsis* reference genome, yielding the mapping ratio between 93.23% and 95.07%, indicating that RNA-seq data were of high quality and reliability. In order to gain a comprehensive view of transcript abundance in *lncRNA18313*-OE and WT plants under 100 μM CdCl_2_ conditions, a heatmap was constructed by clustering all DEGs, highlighting the changes in gene expression levels. In the present study, overexpression of *lncRNA18313* in *Arabidopsis* was found to significantly alter transcript abundance under Cd stress conditions (**Figure 3A**). Compared with WT, *lncRNA18313*-OE can regulate 370 differentially expressed genes with more than a two-fold change in expression under Cd stress (**Figure 3B**). Of these, 203 differentially expressed genes (54.9%) were upregulated, while 167 genes (45.1%) were downregulated. Based on these DEGs, it is likely that *TalncRNA18313* is positively associated with the expression of Cd-response genes.

**Table 1:** Summary of sequence assembly after Illumina sequencing.

Sample	Raw reads	Clean reads	Clean bases	Q20 (%)	Q30 (%)	GC content (%)
WT-1	40239198	39355774	5.90G	99.79	99.85	45.0
WT-2	40522008	39453474	5.92G	99.78	99.80	45.0
WT-3	35468430	34460832	5.17G	99.93	99.42	45.0
OE-1	37608886	36548828	5.48G	99.77	98.78	44.5
OE-2	40799244	39531784	5.93G	99.76	98.81	45.5
OE-3	34753002	33874300	5.08G	99.78	98.83	45.0

WT-1, WT-2 and WT-3: wild-type *Arabidopsis* (three independent biological replicates).

OE-1, OE-2 and OE-3: *lncRNA18313*-OE *Arabidopsis* (three independent biological replicates).

Q20: The percentage of bases with a Phred value >20.

Q30: The percentage of bases with a Phred value >30.

To verify the results of RNA-sequencing, a total of 10 DEGs involved in Cd tolerance regulated by *lncRNA18313* were selected for qRT-PCR analysis. As shown in **Figure 3C**, the expression patterns of these 10 DEGs were in agreement with the RNA-seq results. The value of R square for the RNA-seq vs. qRT-PCR was 0.9132, further confirming the high reliability of identified DEGs in this study.

### 3.4 Potential functional analysis of differentially expressed genes regulated by *lncRNA18313* under Cd stress

**Figure 4:**
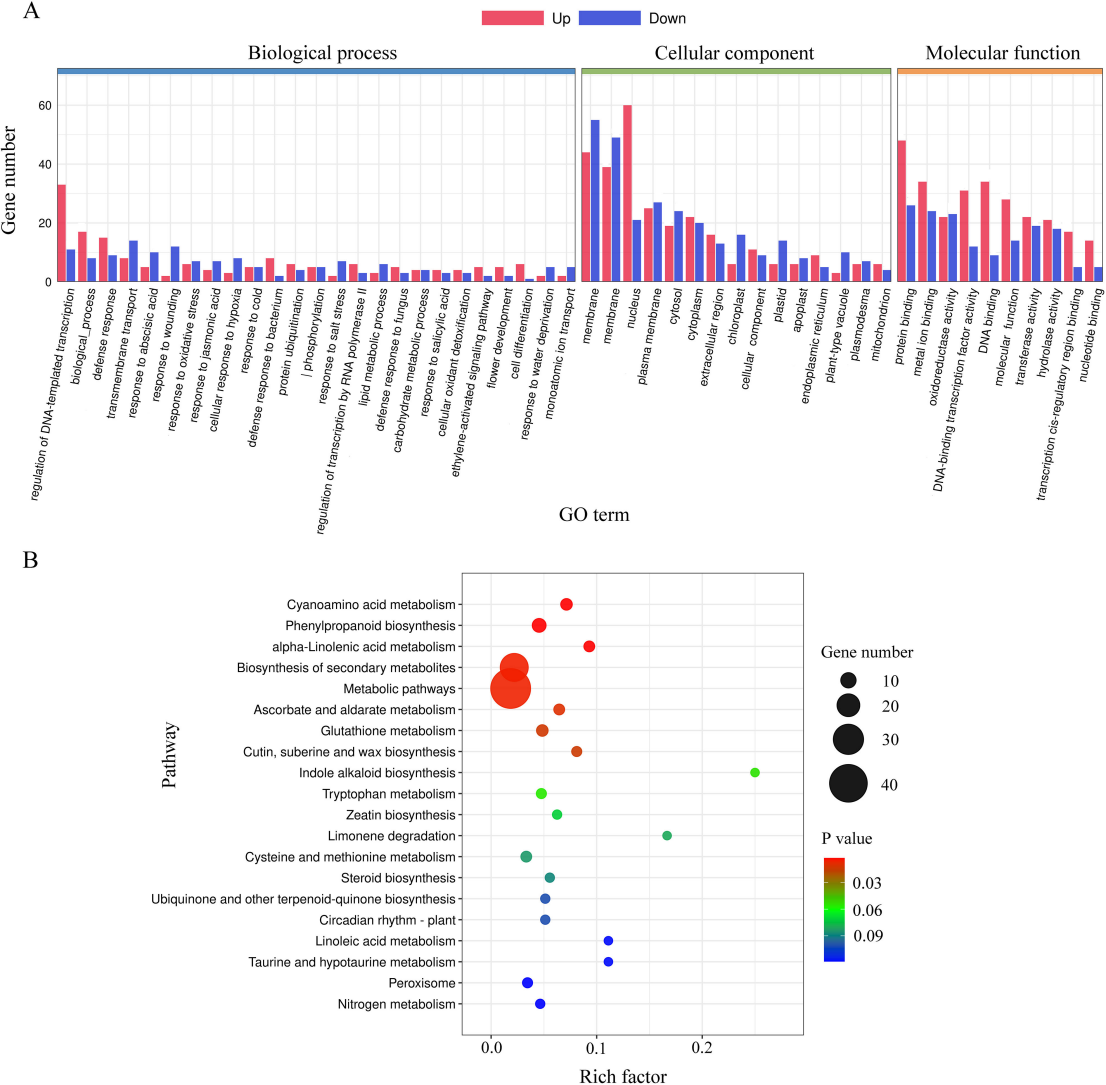
Gene ontology (GO) and Kyoto Encyclopedia of Genes and Genomes (KEGG) enrichment analysis of differentially expressed genes between *lncRNA18313*-OE and WT plants under 100 μM Cd stress. **(A)** GO enrichment analysis. The X-axis represents the GO term, while the Y-axis indicates the number of genes within each category. **(B)** KEGG enrichment analysis. The Y-axis indicates the KEGG pathway, the X-axis indicates rich factor, which is the ratio of differentially expressed gene numbers annotated in this pathway term to all gene numbers annotated in this pathway term. The color of the dot represents p-value, while the size of the dot represents the number of DEGs involved in the respective pathway.

To infer the potential functions of DEGs regulated by *lncRNA18313*, GO term and KEGG pathway enrichment analyses were conducted on these significantly regulated genes. All DEGs were classified into 50 GO terms (**Figure 4A**), including 25 in “biological process” (BP), 10 in “molecular function” (MF), and 15 in “cellular component” (CC) (**Figure 4A**). GO terms associated with responses to various stresses, such as response to salt stress (GO: 0009651), response to water deprivation (GO: 0009414), response to oxidative stress (GO: 0006979) and response to wounding (GO: 0009611) were highly enriched in the biological process category. Molecular functions predominantly highlighted binding and catalytic activity. Additionally, KEGG pathway analysis showed that DEGs regulated by *lncRNA18313* were significantly enriched in 52 KEGG pathways (**Figure 4B**). Notably, metabolic pathways (ath01100, 43), biosynthesis of secondary metabolites pathway (ath01110, 27), phenylpropanoid biosynthesis pathway (ath00940, 8) and glutathione metabolism pathway (ath00480, 5) were particularly enriched in *lncRNA18313*-OE plants under Cd stress based on KEGG pathways. Collectively, these findings suggest that the DEGs regulated by *lncRNA18313* are implicated in a broad array of biological processes and regulatory networks, particularly in response to environmental stressors, such as Cd.

### 3.5 *LncRNA18313* regulates the expression of DEGs involved in antioxidative defense responses

**Figure 5:**
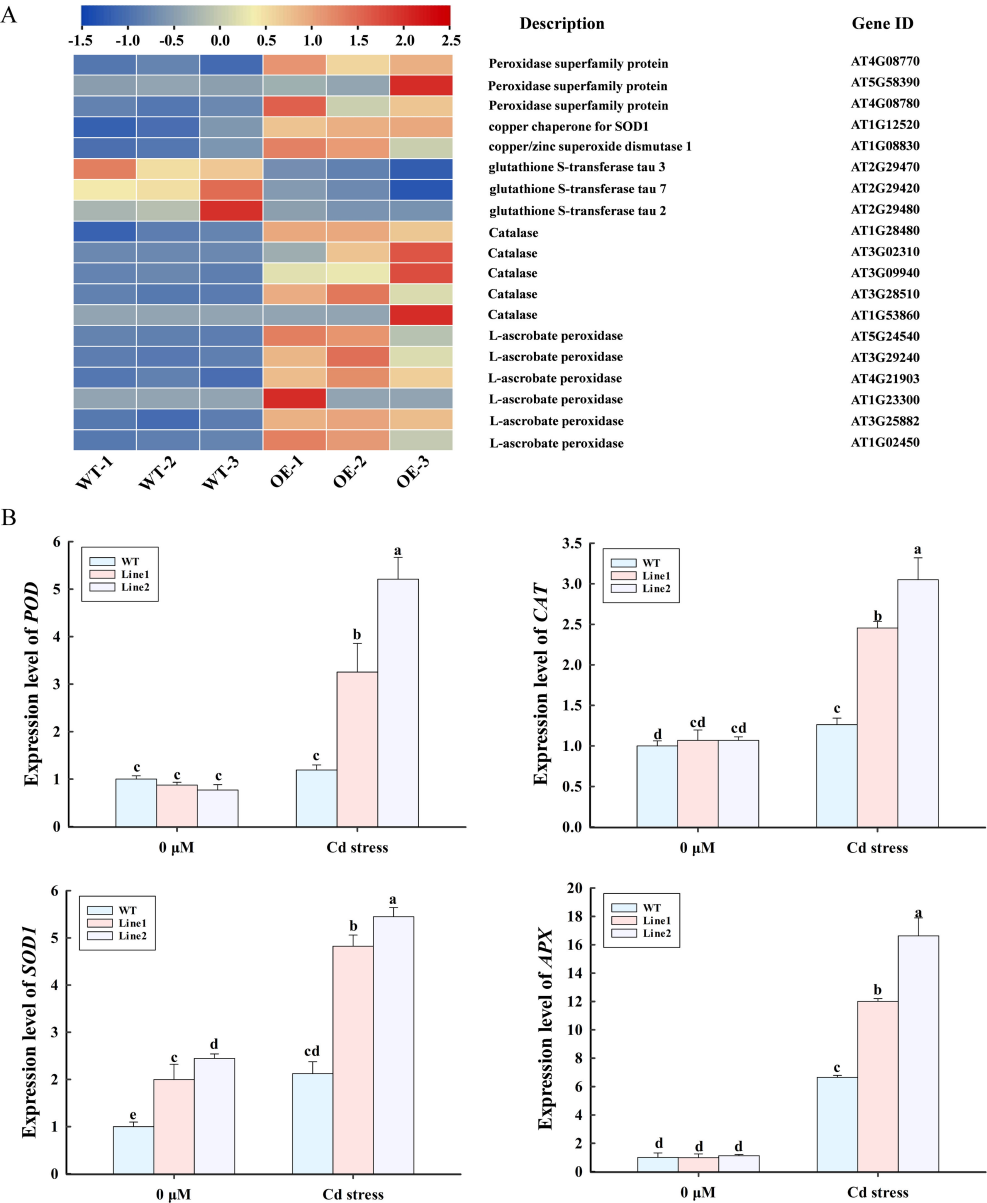
Effects of overexpressing *lncRNA18313* on the expression of differentially expressed genes (DEGs) related to antioxidant enzymes in *Arabidopsis* under Cd stress. **(A)** Heatmap of DEG expressions. The bar represents the scale of the expression level for each gene as indicated by blue (low expression) and red rectangles (high expression). **(B)** RT-qPCR analysis of the expression profiles of four selected transcripts. 0 μM, no Cd treatment; Cd stress, 100 μM CdCl_2_. The bars (means ± SD, n = 3) labeled with different letters indicate significant differences (*p* < 0.05) between treatments as determined by one-way ANOVA with Duncan’s multiple range test.

RNA-Seq analysis indicated that genes associated with *POD*, *SOD*, and glutathione S-transferase (*GST*) were notably regulated in *lncRNA18313*-OE plants under Cd stress (**Figure 5A**). Compared to WT plants, 16 antioxidant enzyme genes were significantly upregulated in *lncRNA18313*-OE plants, including encoding three *PODs* (AT4G08770, AT5G58390, AT4G08780), two *SODs* (AT1G12520, AT1G08830), five catalases (*CATs*) (AT1G28480, AT3G02310, AT3G09940, AT3G28510, AT1G53860), and six ascorbate peroxidases (*APXs*) (AT5G24540, AT3G29240, AT4G21903, AT1G23300, AT3G25882, AT1G02450) (**Figure 5A**). Interestingly, three *GST* (AT2G29470, AT2G29420, AT2G29480) were significantly downregulated in *lncRNA18313*-OE plants compared to WT plants. To validate these findings, we further assessed the expression levels of *POD* (AT4G08770), *APX* (AT1G02450), *SOD1* (AT1G08830), *CAT* (AT1G28480) by RT-qPCR in both WT and *lncRNA18313*-OE plants, with or without Cd treatment. Under normal condition, no dramatic difference were observed in the expression levels of the *POD*, *APX*, *SOD1* and *CAT* between WT and *lncRNA18313*-OE plants. However, under Cd stress, the expression levels of *POD*, *APX*, *SOD1* and *CAT* were significantly higher in the *lncRNA18313*-OE plants (**Figure 5B**). Furthermore, the expression trends of those four genes were also largely consistent with the RNA-Seq data, although minor variations in expression levels were observed.

### 3.6 *LncRNA18313* regulates the expression of DEGs involved in transcriptional regulation

**Figure 6:**
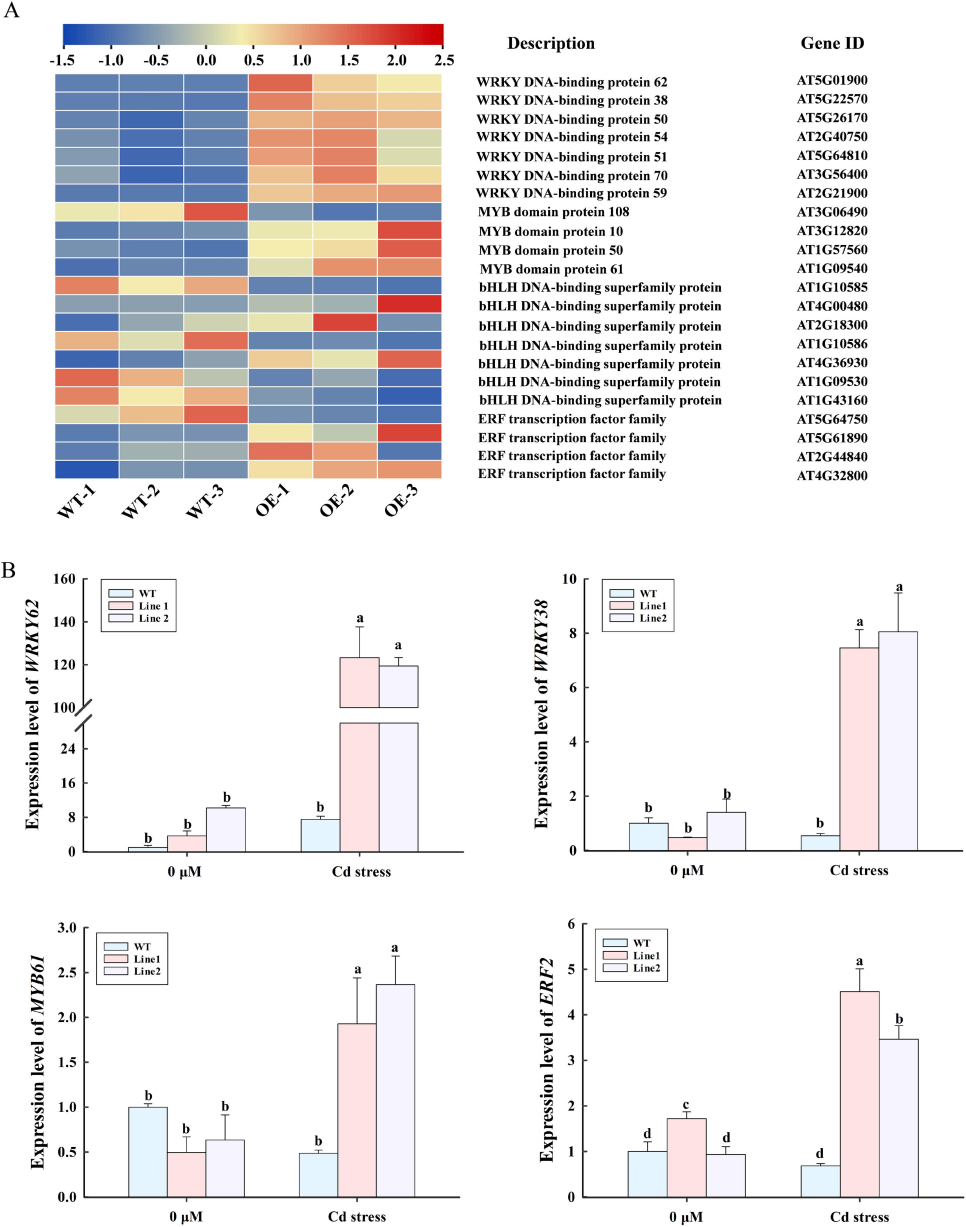
Effects of overexpressing *lncRNA18313* on the expression of DEGs related to transcription factors in *Arabidopsis* under Cd stress. **(A)** Heatmap of DEG expression associated with transcription factors. The color scale represents gene expression levels, with blue indicating low expression and red indicating high expression. **(B)** RT-qPCR analysis of expression profiles in four selected transcripts under Cd stress. 0 μM, no Cd treatment; Cd stress, 100 μM CdCl_2_. Bars represent means ± SD (n = 3), with different letters indicating significant differences (*p* < 0.05) between treatments as determined by one-way ANOVA with Duncan’s multiple range test.

Transcriptome analysis in *lncRNA18313*-OE plants subjected to Cd stress revealed 43 differentially expressed transcription factors (TFs) from 14 TF families, with 35 upregulated and 8 downregulated (**Supplementary Figure S2**). Among them, 22 transcription factors encoding *WRKY*, *MYB*, *bHLH* and *ERF* were potentially involved in abiotic stress tolerance according to their functional annotation (**Figure 6A**). Compared to WT plants, 16 transcription factors genes, including encoding 7 *WRKY* (AT5G01900, AT5G22570, AT5G26170, AT2G40750, AT5G64810, AT3G56400, AT2G21900), 3 *MYB* (AT3G12820, AT1G57560, AT1G09540), 3 *bHLH* (AT4G00480, AT2G18300, AT4G36930), 3 *ERF* (AT5G61890, AT2G44840, AT4G32800) were significantly upregulated in *lncRNA18313*-OE plants under Cd stress (**Figure 6A**). In addition, 6 TF genes, including *MYB* (AT3G06490), *bHLH* (AT1G10585, AT1G10586, AT1G09530, AT1G43160), and *ERF* (AT5G64750), were significantly downregulated in the *lncRNA18313*-OE plants under Cd stress. Subsequently, the expression levels of 3 TFs, *WRKY*, *MYB* and *ERF* in *lncRNA18313*-OE plants under Cd stress were further validated by RT-qPCR. The results demonstrated a clear upregulation of the *WRKY*, *MYB* and *ERF* transcription factors in the *lncRNA18313*-OE plants under Cd stress when compared to the WT plants (**Figure 6B**). These findings suggest that these transcription factors, regulated by *lncRNA18313*, play a key role in mediating plant’s response to Cd-induced oxidative stress.

## 4 Discussion

### 4.1 *LncRNA18313* enhances plant tolerance to Cd stress

With the rapid advancement of RNA-Seq technology, a growing number of lncRNAs have been emerging in various plant species, including key crops such as wheat and barley (Li et al., 2022; Bai et al., 2024). Some plant lncRNAs have been studied intensively, particularly those involved in responses to various stresses, such as cold stress in wheat (Lu et al., 2020) and cotton (Cao et al., 2021), drought stress in *Arabidopsis* (Qin et al., 2017), salt stress in *Ginkgo biloba* (Liu et al., 2025) and cotton (Zhang et al., 2021). However, the biological functions and molecular mechanisms of most lncRNAs still remain elusive, especially those involved in Cd stress in wheat. In our previous study, a Cd-responsive lncRNA, *lncRNA18313* was identified in wheat through RNA sequencing (Zhu et al., 2023). The present study aims to functionally characterize *TalncRNA18313* by overexpressing it in *Arabidopsis*, which lacks a homologous sequence. Because genetic transformation in wheat is still very difficult currently, we failed to generate transgenic wheat in this work. Therefore, we used *Arabidopsis* to overexpress *TalncRNA18313*. In order to explore whether *lncRNA18313* is associated with plant tolerance to Cd stress, 6-week-old wild-type (WT) and *TalncRNA18313*-overexpressing *Arabidopsis* seedlings were incubated under 100 μM CdCl_2_ conditions in growth chambers. After 4 days of Cd stress, WT plants exhibited more severe damage compared to the *lncRNA18313*-OE *Arabidopsis* seedlings. Specifically, most of WT plant leaves were completely withered and dry, while the *lncRNA18313*-OE plants showed only slight damage, with fewer wilted leaves and a relatively healthy appearance compared to the WT. These results suggest that overexpression of *TalncRNA18313* confers greater tolerance to Cd stress, similar to the function of other lncRNAs, such as *lncRNA37228* in wheat (Zhu et al., 2023) and *MSTRG.22608.1* in *Populus tomentosa* (Quan et al., 2021). To further characterize how *TalncRNA18313* enhances Cd tolerance in *Arabidopsis*, the indicator of oxidative damage, malondialdehyde (*MDA*) was firstly measured. In the current study, *MDA* content increased significantly in WT plants under Cd stress. However, *lncRNA18313*-OE plants had slightly lower *MDA* content under Cd stress, indicating less oxidative damage to the membrane system. A similar reduction in *MDA* content was observed in DRIR (drought-induced lncRNA) overexpressing *Arabidopsis* plants under drought stress (Qin et al., 2017). Also, overexpression of wheat *lncRNA37228* in *Arabidopsis* plants resulted in lower *MDA* content comparing to WT plants under 150 μM Cd treatment for 7 days (Zhu et al., 2023). Plants eliminate excessive reactive oxygen species (ROS) and mitigate oxidative damage by regulating the activities of key antioxidant enzymes, such as *SOD*, *CAT* and *POD* (Zhu et al., 2023; Wang et al., 2024). In this study, the activities of *SOD*, *POD* and *CAT* in the *lncRNA18313*-OE plant were significantly higher than those observed in WT plants under Cd stress. This result aligns with findings reported by Lu et al. (2020), who showed that overexpression of wheat *lncRNA117* led to a significant increase in the activities of *SOD* and ascorbate peroxidase (*APX*) in transgenic *Arabidopsis* plants exposed to low-temperature stress. Similarly, Jia et al. (2024) demonstrated that overexpression of long non-coding RNA *lncSIR1* from *Betula platyphylla* enhanced salt tolerance in transgenic *Arabidopsis* by boosting the activities of *CAT*, *POD*, and *APX* during salt stress for 21 days. Therefore, the increased activities of *SOD*, *POD*, and *CAT* in *lncRNA18313*-OE plants may play a crucial role in reducing oxidative damage, thereby contributing to improve Cd tolerance in *Arabidopsis*.

### 4.2 Regulation of transcription factors by *lncRNA18313* confers Cd tolerance

GO enrichment analysis in the present study revealed that DEGs associated with response to oxidative stress were significantly enriched in *lncRNA18313*-OE plants under Cd stress compared to WT plants. Transcription factors (TFs) are critical in wheat response to various abiotic stresses, such as heavy metal (Zhu et al., 2022), salt (Wei et al., 2017) and drought (Xue et al., 2011), as demonstrated over the last decade. Many TFs from various families, such as *ERF* (ethylene responsive element binding protein), *WRKY*, *bHLH* (basic helix-loop-helix) and *MYB* (Myb-like DNA-binding domain) were considered as key players of transcriptional changes involved in plant stress adaptation response (Gao et al., 2016; Sheng et al., 2019). Li et al. (2021) studied the overexpression of *CRIR1*, a cold‐responsive intergenic *lncRNA 1*, in cassava and found that it positively modulated cold tolerance through upregulating *MeNAC* transcription factors. It was recently demonstrated that *NAC* transcription factors played a significant role in various abiotic stress responses (Shao et al., 2015). Overexpressing *NAC* in *Arabdopsis* has been found to confer salt stress in maize (Pan et al., 2024) and drought stress in wheat (Xue et al., 2011). Additionally, long non-coding RNA, *LncY1* from birch improves salt tolerance by upregulating *BpMYB96* transcription factors (Jia et al., 2023). Overexpression of *MYB56* isolated from wheat, improved salt and freezing stress tolerance, accompanied by higher activities of *POD*, *CAT* and *SOD* in transgenic *Arabidopsis* (Zhang et al., 2012). In this study, sixteen transcription factors genes, including encoding seven *WRKY*, three *MYB*, three *bHLH*, three *ERF* were significantly upregulated in the *lncRNA18313*-OE plants exposed to Cd stress when compared to the WT plants. In birch (*Betula platyphylla*), overexpression of long noncoding RNA, *BplncSIR1* in *Arabidopsis* enhanced salt tolerance by upregulating *BpNAC2* to mediate ROS scavenging (Jia et al., 2024). In line with this, the RT-qPCR analysis further revealed a significant induction of *WRKY*, *MYB* and *ERF* transcripts in *lncRNA18313*-OE plants exposed to Cd stress. As reported previously, transgenic *Arabidopsis* overexpressing the *TaMYB73*, *TaNAC29* and *TaWRKY44* transcription factor genes from wheat, respectively significantly improved salt (He et al., 2012), drought (Huang et al., 2015) and cold (Wang et al., 2015) stress tolerance. Thus, the elevated transcriptional levels of *NAC*, *WRKY* and *MYB* transcription factor genes regulated by *lncRNA18313* were likely to contribute to the increase tolerance of wheat seedlings to Cd stress. However, additional work is needed to test this possibility and to fully elucidate the function of *lncRNA18313*.

## 5 Conclusion

In this research, we functionally characterized the wheat long non-coding RNA, *lncRNA18313*, by overexpressing it in *Arabidopsis*. Through phenotypic, physiological, and transcriptomic analyses conducted in this work, we demonstrated that *lncRNA18313* overexpression enhances Cd tolerance in the transgenic plants by modulating the expression of transcription factors to mediate ROS scavenging. However, our understanding of the complex regulatory role of *lncRNA18313* is still in its infancy, and more attempts are still required to unravel its regulatory mechanism in the future.

## Data Availability

The datasets presented in this study can be found in online repositories. The names of the repository/repositories and accession number(s) can be found in the article/**Supplementary Material**.
