# Supplementary material for: A long non-coding RNA lncRNA18313 regulates resistance against cadmium stress in wheat
Source: Front Plant Sci. 2025 Jun 2;16:1583758. doi: 10.3389/fpls.2025.1583758 (PMC12171198; doi:10.3389/fpls.2025.1583758)
Supplement: Supplementary file 1 [file DataSheet1.docx]

Fig.S1 The expression levels of *lncRNA18313* in wheat leaves at different time points under normal growth conditions. *lncRNA18313* expression was measured by qRT-PCR and normalized against the *TaEF1* gene. Each bar represents the mean ± standard error of six replicates. Columns labeled with different letters indicate a significant difference (p *<*0.05), as determined by Duncan's multiple range test.

Fig.S2 Effects of overexpressing *lncRNA18313* on the expression of differentially expressed transcription factors (TFs) in *Arabidopsis* under Cd stress

**Table S1 Primers used for qRT-PCR analysis**

| **Primer use** | **Gene name** | **Primer sequence (5’–3’)** |
| --- | --- | --- |
| RT-qPCR | *Lnc18313-*QF | TGCGCGGTACAAAGTTCCT |
|  | *Lnc18313-*QR | GGGTTTCATTTGGATTCCCCT |
|  | *TaEF1-*QF | TGGTGTCATCAAGCCTGGTATGGT |
|  | *TaEF1-*QR | ACTCATGGTGCATCTCAACGGACT |
|  | *Actin2-*QF | ACCTTGCTGGACGTGACC |
|  | *Actin2-*QR | TCCCGCTCTGCTGTTGTG |
|  | *WRKR62-*QF | CCACCTCTAACCTCCGCC |
|  | *WRKY62-*QR | GGCAACCTCGGACGACAT |
|  | *WRKY38-*QF | CACACGAAAAGGCGGTGC |
|  | *WRKY38-*QR | AAGCGGTCCACCATCAGC |
|  | *MYB61-*QF | TAGCCCCGGGAACATGGT |
|  | *MYB61-*QR | TCCCAGTTTGGTGCAGCC |
|  | *ERF2-*QF | GCGGTGGGTATGGGAGTG |
|  | *ERF2-*QR | TCCCCAAAGCGCCAGAAT |
|  | POD-QF | ATGAAGGCTGCCGTGGAG |
|  | POD-QR | GACCTCCCGCCAAAACGA |
|  | CAT-QF | TTGACCGCCGCAGTGATT |
|  | CAT-QR | TGGCCACGAAATCGCTGT |
|  | APX-QF | GTCGGGGAATCTCTGCGG |
|  | APX-QR | GCGTTCTCCTCCACCACC |
|  | SOD1-QF | GGGCTGTTGTTGTCCATGC |
|  | SOD1-QR | CCGCCTGCGTTTCCAGTA |
